# Supplementary material for: Analysis of PPARγ Signaling Activity in Psoriasis
Source: Int J Mol Sci. 2021 Aug 10;22(16):8603. doi: 10.3390/ijms22168603 (PMC8395241; doi:10.3390/ijms22168603)
Supplement: Supplementary file 1 [file ijms-22-08603-s001.zip › Supplemental materials_Analysis of PPARg signaling activity in psoriasis/Pathway models/Models images and html files/PPARG expressed in cells assotiated with psoriasis/213412.html]

immune system


# Organ immune system

|  |  |
| --- | --- |
| URN | urn:agi-ncimorgan:C0020962 |
| Total Entities | 4 |
| Connectivity | 8193 |
| Name | immune system |

---

|  |  |
| --- | --- |
| ChildConcepts | lymphoid tissue |
|  | lymphoid organ |
|  | immune system cell |
|  | reticuloendothelial system |

---

|  |  |
| --- | --- |
| Pathway | PPARG expressed in cells assotiated with psoriasis |
|  | Neighbors of immune system |

---

|  |  |
| --- | --- |
| MedScan ID | 8802958 |

---

|  |  |
| --- | --- |
| Alias | immune system |
|  | allergic/immunologic organ system |
|  | immune systems |
|  | allergic/immunologic body system |

---

|  |  |
| --- | --- |
| NCIm ID | C0020962 |

---
